# Neanderthal language? Just-so stories take center stage

**DOI:** 10.3389/fpsyg.2013.00671

**Published:** 2013-09-24

**Authors:** Robert C. Berwick, Marc D. Hauser, Ian Tattersall

**Affiliations:** ^1^Department of Electrical Engineering and Computer Science/Brain and Cognitive Sciences, Laboratory for Information and Decision Systems, Massachusetts Institute of TechnologyCambridge, MA, USA; ^2^Risk-Eraser, LLCFalmouth, MA, USA; ^3^Division of Anthropology, American Museum of Natural HistoryNew York, NY, USA

**Keywords:** language evolution, human evolution, language contact, genetic admixture

Dediu and Levinson (2013) link two extraordinary claims: first, humans and Neanderthals were one and the same species and second, “Speech and language … are ancient, being present in a modern-like form over half a million years ago in the common ancestor of Neanderthals and modern humans, the result of evolution in the prior one million years or so as *H. heidelbergensis* evolved from *H. erectus*” (p. 12). These claims are marred by their selective review of the literature; the use of equivocal evidence as definitive support for their interpretation; and the lack of any evolutionary evidence regarding the computations and representations that mediate modern linguistic competence.

Our language phenotype is a competence consisting of three systems—syntax, semantics, and phonology—that are internal to the mind/brain, along with two mediating interfaces, the first linking the representational/computational systems to external speech or sign, the second linking language to internal thought. The internal combinatorial computations are generative and rule-governed, engaging representations that are both language-specific and domain-general. Generalized claims such as Dediu and Levinson's require evidence for all of these processes. But most of them do not leave fossil evidence; and genetic clarification is unlikely given how poorly we understand simpler phentoypes. For example, though language is expressed through articulate speech today, the anatomical capacity for speech cannot by itself be taken as a proxy for language. The peripheral organs have to connect with the *internal* phonological, syntactic and semantic representations, and nothing in the fossil record is ever likely to tell us: (a) what those representations were like; or (b) whether the human brain had yet formed the necessary connections between, e.g., phonological representations and vocal output. This does not bode well for the type of argument Dediu and Levinson advance, but let us examine the evidence they present.

Dediu and Levinson never describe the language phenotype, except to note that it is the “full suite of abilities to map sounds to meaning” (p. 2). Because the phenotype is not specified at the required level of detail for evolutionary analysis, it is difficult to test their evolutionary claims. But this lack of detail does permit them to assert that language competence was likely among Neanderthals, due to nothing more than their large brains, cognitive complexity, and proclaimed conspecificity with modern humans. Still, hominids can be smart without implying modern cognition (Tattersall, 2012), and smart does not necessarily mean that Neanderthals had the competence for language or the capacity to externalize it in speech. Further, while Dediu and Levinson rely on recent reports of low-level genetic interchange between *Homo neanderthalensis* and *Homo sapiens* as evidence of conspecificity, minor gene flow is to be expected between closely related sympatric species (Coyne and Orr, 2004; Mallet, 2005), and anatomical and genetic analyses reveal that both lineages were morphologically and historically individuated (Tattersall and Schwartz, 2006; Currat and Excoffier, 2011). Their argument additionally rests on the implicit claim that limited interbreeding among these hominids, with resulting genetic admixture, actually yielded specific genetic differences in African vs. non-African human populations that led to *specifically different phenotypic language traits*. But no evidence is provided in support of this claim.

Dediu and Levinson assert that unambiguous symbolic behavior among the Neanderthals/Denisovans provides evidence of language competence. There are two grave problems with this claim: symbolic behavior, as in cave painting, is an indirect proxy for language, and its earliest indications come from *Homo sapiens*, not Neanderthals, and only at sites dated at roughly 100 kyr or less [e.g., 77-kyr engraved ochres from Blombos Cave, South Africa; (Henshilwood et al., 2009)]. Archaeology thus supports a recent timeframe for the emergence of modern behaviors associated with language: substantially *after* the emergence of *Homo sapiens* at ca. 200 kyr. But even this evidence is silent on semantic representations, links to syntax and phonology, and so on. Thus, even if *all* of their supposed evidence were germane and correct, it would not suffice to demonstrate that either Neanderthals—or even the first *Homo sapiens*—had modern language competence, or the capacity to externalize it in speech.

Along with the (expected) genomic similarity among Neanderthals, Denisovans and modern humans, Dediu and Levinson present several gene-level commonalities as evidence for Neanderthal genomic compatibility with language, e.g., *FOXP2*. Again, the analysis is highly selective. *FOXP2* is only one of many genes contributing to articulate speech. It has little to do with the central control of language, but is rather involved with its externalization. Neither Neanderthals nor Denisovans possessed human variants of other putatively “language-related” alleles such as *CNTAP2*, *ASPM*, and *MCPH1* (*Microcephalin*), among others, so the evidence is actually equivocal. The survey they themselves cite (Somel et al., 2013) arrives at a different conclusion: “There is accumulating evidence that human brain development was *fundamentally reshaped* through several genetic events within the short time space *between the human-Neandertal split and the emergence of modern humans*” (Somel et al., 2013, p.119, our emph.). In any event, it is currently unknown how genetic variants build language competence in modern humans, let alone in Neanderthals.

Dediu and Levinson conclude that (1) “saltationist” stories for language origin must be rejected; (2) language evolved incrementally; (3) gene-culture interaction greatly affected language's “design”; (4) a “deeper” historical linguistics is called for; and (5) modern human languages might not reflect the full space of language possibilities. All this is speculative to the point of irrelevance. (1) is contradicted both by the modern understanding of evolutionary patterns (Thompson, 2013) and by empirical evidence that hominid evolution is best characterized by long periods of stasis interspersed with more rapid changes (Tattersall, 2012); no evidence whatsoever is brought forward to support (2); (3) and (4) are directly contradicted both by the rapidity of gene-culture co-evolution that they themselves embrace (Baronchelli et al., 2012) and also by psycholinguistic findings (Wong et al., 2012); and (5) is contradicted by evidence that the space of possible phonological and syntactic variation has not really changed in the past 10 kyr—Ancient Sumerian looks just like any other contemporary human language.

In short, Dediu and Levinson's extraordinary claims are not supported by the evidence they present. More significantly, we doubt that there *could* be any evidence to support such claims. At the archaeological level, our core linguistic competence does not fossilize. As for molecular evidence, we are nowhere near identifying the relevant “language genotype” and they provide no “language phenotype” to guide us. For the present, abstinence from speculation may be the best remedy.

## Author contributions

Robert C. Berwick, Marc D. Hauser, and Ian Tattersall contributed equally to the writing of this commentary.
